# Modulation of the cortical silent period elicited by single- and paired-pulse transcranial magnetic stimulation

**DOI:** 10.1186/1471-2202-14-43

**Published:** 2013-04-02

**Authors:** Sho Kojima, Hideaki Onishi, Kazuhiro Sugawara, Hikari Kirimoto, Makoto Suzuki, Hiroyuki Tamaki

**Affiliations:** 1 Graduate school of Health and Welfare, Niigata University of Health and Welfare, Nigata, Japan; 2 Institute for Human Movement and Medical Sciences, Niigata University of Health and Welfare, 1398 Shimami-cho, Kita-ku, Niigata City, Niigata, 950-3198, Japan

**Keywords:** Transcranial magnetic stimulation, Motor evoked potential, Cortical silent period, Short-interval intracortical inhibition, Intracortical facilitation

## Abstract

**Background:**

The cortical silent period (CSP) elicited by transcranial magnetic stimulation (TMS) is affected by changes in TMS intensity. Some studies have shown that CSP is shortened or prolonged by short-interval intracortical inhibition (SICI) and intracortical facilitation (ICF), Those studies, however, used different TMS intensities to adjust the amplitude of the motor evoked potential (MEP). Therefore, it is unclear whether changes in CSP duration are induced by changes in TMS intensities or by SICI and ICF. The purpose of this study was to confirm the effects of muscle contractions and stimulus intensities on MEP amplitude and the duration of CSP induced by single-pulse TMS and to clarify the effects of SICI and ICF on CSP duration.

MEP evoked by TMS was detected from the right first dorsal interosseous muscle in 15 healthy subjects. First, MEP and CSP were induced by single-pulse TMS with an intensity of 100% active motor threshold (AMT) at four muscle contraction levels [10%, 30%, 50%, and 70% electromyogram (EMG)]. Next, MEP and CSP were induced by seven TMS intensities (100%, 110%, 120%, 130%, 140%, 150%, and 160% AMT) during muscle contraction of 10% EMG. Finally, SICI and ICF were recorded at the four muscle contraction levels (0%, 10%, 30%, and 50% EMG).

**Results:**

MEP amplitudes increased with increases in muscle contraction and stimulus intensity. However, CSP duration did not differ at different muscle contraction levels and was prolonged with increases in stimulus intensity. CSP was shortened with SICI compared with CSP induced by single-pulse TMS and with ICF at all muscle contraction levels, whereas CSP duration was not significantly changed with ICF.

**Conclusions:**

We confirmed that CSP duration is affected by TMS intensity but not by the muscle contraction level. This study demonstrated that CSP is shortened with SICI, but it is not altered with ICF. These results indicate that after SICI, CSP duration is affected by the activity of inhibitory intermediate neurons that are activated by the conditioning SICI stimulus.

## Background

Motor evoked potential (MEP) is recorded from peripheral muscles after stimulation of the primary motor cortex by transcranial magnetic stimulation (TMS). TMS produces MEP followed by a period of electromyogram (EMG) silence during voluntary muscle contraction. This period of silence is known as the cortical silent period (CSP). While spinal mechanisms may be active in the early part (approximately 50 ms) of CSP, the cortical origin of at least the later part (approximately 100 ms) of CSP has been proven in several previous studies [1-5]. MEP amplitude fluctuates with TMS intensity, muscle contraction [6-8], and the difficulty of the task [9]. Several studies have investigated the relationship between MEP amplitude and CSP duration; however, this relationship remains unclear. For example, Wu et al. [10] reported that a large MEP amplitude induces CSP for a long duration, whereas Gilio et al. [11] found that CSP duration was not related to the MEP amplitude.

Recently, paired-pulse TMS has become a useful tool for testing cortical inhibition or facilitation of the human motor cortex. When a subthreshold conditioning pulse (S1) and a suprathreshold test pulse (S2) were applied to the motor cortex through the same coil, MEP evoked by the test pulse was inhibited at interstimulus intervals (ISI) between 1 and 5 ms [12]. This phenomenon is known as short-interval intracortical inhibition (SICI) [12-14]. The mechanism of SICI was reported to result from synaptic interaction occurring within M1, and it appeared to be mediated at the cortical level [12,15]. Therefore, a subthreshold S1 suppressed the size of both the descending spinal cord volleys and MEP induced by a suprathreshold S2 [16]. In pharmacological research, the mechanism of SICI has been explored in more detail, and the involvement of gamma-aminobutyric acid (GABAA) has been suggested [14,17-20]. In contrast, MEP is facilitated at ISI greater than 10 ms, which is known as intracortical facilitation (ICF) [12-14]. Because the intensity of the conditioning stimulus was too weak for enhancement of any effects on spinal H reflexes, it was considered that ICF occurred within the cerebral cortex [12,14]. However, Di Lazzaro et al. [21] suggested the possibility that the conditioning stimulus reflects the excitability of spinal motoneurons. Although there is evidence for the origin of SICI, there is less direct information on the origin of ICF.

There have been some studies concerning CSP duration after SICI or ICF during muscle contraction [22-24], whereas many studies of SICI or ICF have been conducted under resting conditions [15,25,26]. MEP amplitude induced by paired-pulse TMS was reported to change during muscle contraction at 5%–50% of maximum voluntary contraction (MVC) [19,25]. Some studies showed that CSP was shortened or prolonged by paired-pulse TMS [22-24]; however, these studies used different intensities of paired-pulse TMS to adjust the MEP amplitude. Therefore, it is unclear whether changes in CSP duration are induced by the change in magnetic stimulation intensities or by SICI or ICF.

The purpose of this study was to confirm the effects of muscle contractions and stimulus intensities on MEP amplitude and the duration of CSP induced by single-pulse TMS and to clarify the effects of SICI and ICF on CSP duration.

## Results

**Figure 1:**
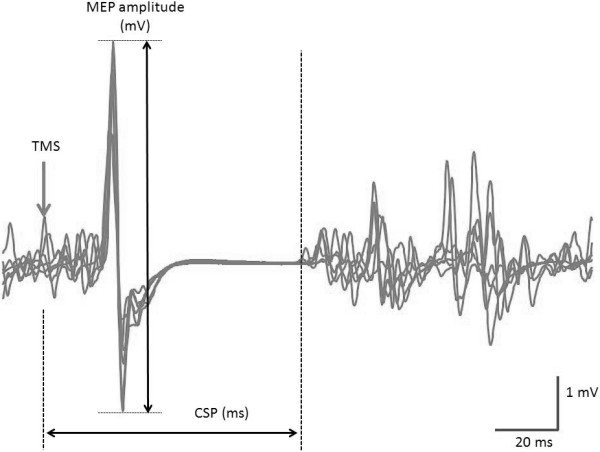
**Representative waveforms of MEP induced by TMS during sustained 30% EMG.** MEP amplitude was calculated by averaging peak-to-peak amplitudes. CSP duration was defined as the time from TMS onset to the time of reappearance of an EMG amplitude that was 3-fold the standard deviation of the background EMG noise at rest.

The 1 mV TMS intensity and active motor threshold (AMT) were 56.5 ± 7.5% and 36.0 ± 4.7% [mean ± standard deviation (SD)], respectively, of the maximum stimulator output. Figure 1 shows representative waveforms of MEP during muscle contraction of 30% EMG. We were able to clearly observe CSP for all muscle contraction levels and all TMS intensities.

### Effects of muscle contraction on MEP amplitude and CSP duration (experiment 1)

**Figure 2:**
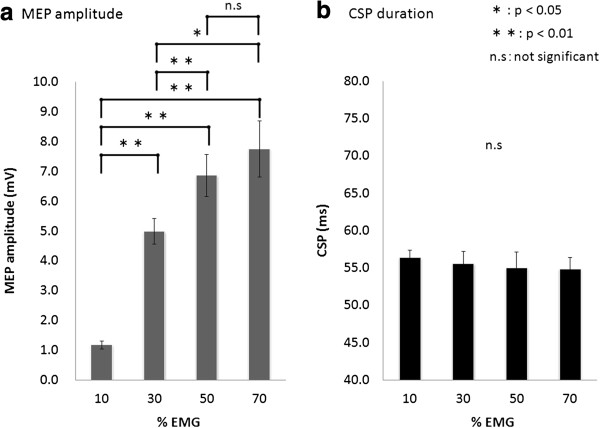
**The effects of voluntary muscle contraction on MEP amplitude and CSP duration.** (**a**) The MEP amplitude induced at 30%, 50%, and 70% EMG was significantly larger than that induced at 10% EMG. The MEP amplitude induced at 50% and 70% EMG was significantly larger than that induced at 30% EMG. (**b**) CSP duration was not significantly influenced by background contraction.

Results of one-way analysis of variance (ANOVA) showed that MEP amplitude changed significantly with increased muscle contraction (F_(3,30)_ = 45.758, p < 0.01; Figure 2a). The MEP amplitude [mean ± standard error of the mean (SEM)] was 1.17 ± 0.13 mV at 10% EMG, 4.98 ± 0.43 mV at 30% EMG, 6.86 ± 0.70 mV at 50% EMG, and 7.75 ± 0.94 mV at 70% EMG. The MEP amplitude at 30%, 50%, and 70% EMG was significantly larger than that at 10% EMG (p < 0.01). Furthermore, the MEP amplitude at 50% and 70% EMG was significantly larger than that at 30% EMG (30% EMG, p < 0.01; 50% EMG, p < 0.05). No significant difference in the MEP amplitude was observed between 50% and 70% EMG.

CSP duration [mean ± standard error of the mean (SEM)] was 56.3 ± 1.0 ms at 10% EMG, 55.5 ± 1.6 ms at 30% EMG, 55.0 ± 2.1 ms at 50% EMG, and 54.8 ± 1.6 ms at 70% EMG. No significant differences in CSP duration were observed among the muscle contraction levels (F_(2.134,21.342)_ = 0.466, p > 0.05; Figure 2b).

### Effects of TMS intensity on MEP amplitude and CSP duration (experiment 2)

**Figure 3:**
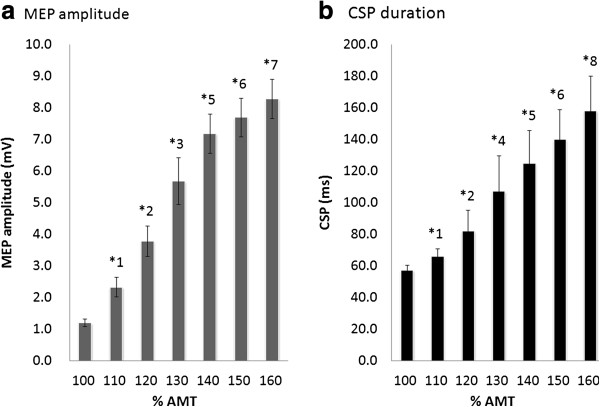
**The effects of different stimulation intensities on MEP amplitude and CSP duration at 10% EMG.** (**a**) The MEP amplitude increased significantly as the stimulus intensity increased from 100% to 140% AMT. However, at stimulus intensities >140% AMT, no significant differences were observed in the MEP amplitudes. (**b**) CSP duration increased significantly as the stimulus intensity increased from 100% to 160% AMT. *1: 110% AMT > 100% AMT (p < 0.01). *2: 120% AMT > 100% AMT, 110% AMT (p < 0.01). *3: 130% AMT > 100% AMT, 110% AMT, 120% AMT (p < 0.01). *4: 130% AMT > 100% AMT, 110% AMT (p < 0.01). *5: 140% AMT > 100% AMT, 110% AMT, 120% AMT, 130% AMT (p < 0.01). *6: 150% AMT > 100% AMT, 110% AMT, 120% AMT, 130% AMT (p < 0.01). *7: 160% AMT > 100% AMT, 110% AMT, 120% AMT, 130% AMT (p < 0.01). *8: 160% AMT > 100% AMT, 110% AMT, 120% AMT, 130% AMT, 140% AMT, 150% AMT (p < 0.01).

Results of one-way ANOVA showed that MEP amplitude changed significantly with increases in TMS intensity (F_(2.356,23.564)_ = 67.687, p < 0.01; Figure 3a). The MEP amplitude at 10% EMG was 1.19 ± 0.13 mV at 100% AMT, 2.32 ± 0.33 mV at 110% AMT, 3.78 ± 0.50 mV at 120% AMT, 5.67 ± 0.77 mV at 130% AMT, 7.18 ± 0.66 mV at 140% AMT, 7.69 ± 0.64 mV at 150% AMT, and 8.28 ± 0.64 mV at 160% AMT. No significant difference in the MEP amplitude was observed at high-output stimulation intensities of >140% AMT.

Results of one-way ANOVA showed that CSP duration changed significantly with increases in TMS intensity (F_(6, 60)_ = 119.578, p < 0.01; Figure 3b). The mean CSP duration was 57.1 ± 1.0 ms at 100% AMT, 65.6 ± 1.6 ms at 110% AMT, 81.7 ± 4.2 ms at 120% AMT, 107.0 ± 7.1 ms at 130% AMT, 124.8 ± 6.6 ms at 140% AMT, 139.9 ± 6.0 ms at 150% AMT, and 157.8 ± 7.0 ms at 160% AMT. CSP duration was prolonged with increasing stimulus intensity. CSP duration at 160% AMT was significantly prolonged compared with those at all other stimulus intensities.

### Effects of paired-pulse TMS on MEP amplitude and CSP duration (experiment 3)

**Figure 4:**
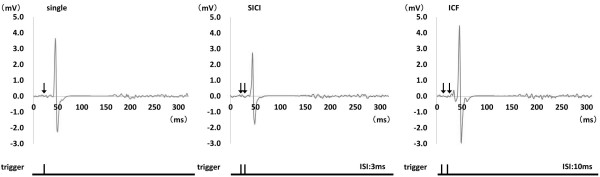
**Representative waveforms of MEP induced by single- and paired-pulse TMS during sustained 10% EMG.** The left panel shows MEP induced by single-pulse TMS. The center panel shows MEP induced by paired-pulse TMS at ISI of 3 ms (SICI) and that the MEP amplitude is smaller than that induced by single-pulse TMS. The right panel shows MEP induced by paired-pulse TMS at ISI of 10 ms (ICF) and that the MEP amplitude is larger than that induced by single-pulse TMS.

**Figure 5:**
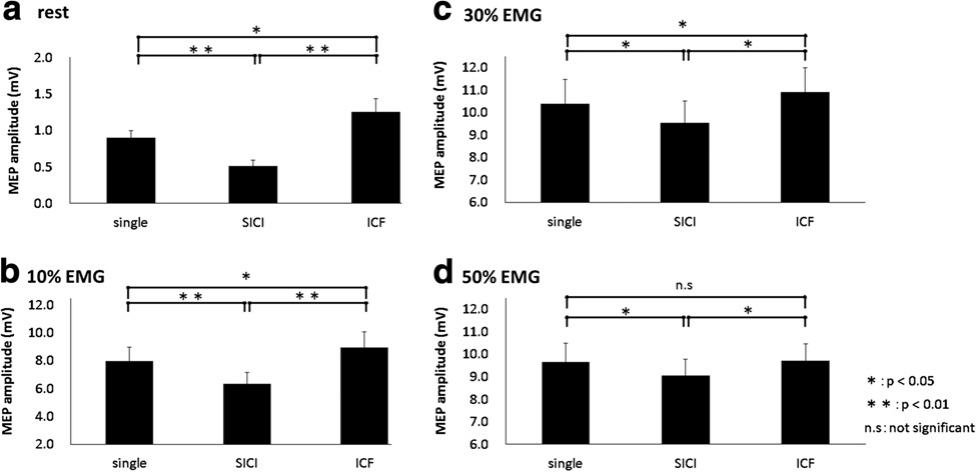
**Histograms of the mean MEP amplitudes obtained with TMS at varying levels of muscle contraction.** At 10% and 30% EMG, as well as at rest, MEP amplitudes induced by SICI were significantly reduced and those induced by ICF were significantly increased compared with those induced by single-pulse TMS (p < 0.05). At 50% EMG, the MEP amplitude induced by SICI was significantly reduced compared with that induced by single-pulse TMS (p < 0.05); however, the MEP amplitude induced by ICF was not significantly increased.

The mean (± SD) conditioning pulse intensity was 32.5 ± 5.8%, and the test pulse intensity was 56.5 ± 7.5%. Figure 4 shows the representative waveforms of MEP induced by paired-pulse TMS at 10% EMG. Results of one-way ANOVA showed that MEP amplitude was significantly changed by paired-pulse TMS (rest, F_(2,20)_ = 18.712, p < 0.01; 10% EMG, F_(2,20)_ = 12.263, p < 0.01; 30% EMG, F_(1.785,17.845)_ = 21.738, p < 0.01; 50% EMG, F_(1.770,17.698)_ = 6.788, p < 0.05). The mean (± SEM) MEP amplitude at rest was 0.90 ± 0.10 mV (single-pulse TMS), 0.51 ± 0.08 mV (SICI), and 1.25 ± 0.18 mV (ICF). The MEP amplitude induced by SICI was significantly smaller than that induced by single-pulse TMS (p < 0.01). Furthermore, the MEP amplitude induced by ICF was significantly larger than that induced by single-pulse TMS (p < 0.05) (Figure 5a). The MEP amplitude at 10% EMG was 7.96 ± 1.02 mV (single-pulse TMS), 6.33 ± 0.84 mV (SICI), and 8.95 ± 1.14 mV (ICF) (Figure 5b) and that at 30% EMG was 10.40 ± 1.09 mV (single-pulse TMS), 9.53 ± 0.98 mV (SICI), and 10.90 ± 1.10 mV (ICF) (Figure 5c). At both 10% and 30% muscle contraction levels, the MEP amplitude induced by SICI was significantly smaller than that induced by single-pulse TMS and ICF (p < 0.05). Furthermore, the MEP amplitude induced by ICF was significantly larger than that induced by single-pulse TMS at 10% and 30% muscle contraction levels (p < 0.05). The MEP amplitude at 50% EMG was 9.67 ± 0.82 mV (single-pulse TMS), 9.06 ± 0.74 mV (SICI), and 9.73 ± 0.74 mV (ICF), and the MEP amplitude induced by SICI was significantly smaller than that induced by single-pulse TMS and ICF (p < 0.05). In contrast, no significant differences were observed in the MEP amplitude induced by single-pulse TMS and ICF (Figure 5d).

**Figure 6:**
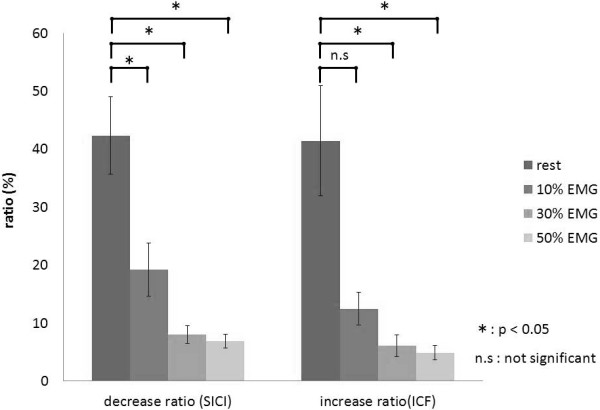
**The effects of voluntary muscle contraction on the decrease and increase ratios of MEP amplitudes.** Among the decrease ratios, the ratio at rest was significantly higher than those at all muscle contraction levels (p < 0.05), and no significant differences were observed at all muscle contraction levels. In contrast, among the increase ratios, the ratio at rest was significantly higher than those at muscle contractions of 30% and 50% EMG (p < 0.05), and no significant differences were observed at all muscle contraction levels.

Results of one-way ANOVA showed that the decrease and increase ratios changed significantly with increases in muscle contractions (decrease ratios, F_(3,30)_ = 27.744, p < 0.01; increase ratios, F_(1.253,12.530)_ = 11.571, p < 0.01; Figure 6). The decrease ratios with SICI were 42.3 ± 6.7% (rest), 19.2 ± 4.6% (10% EMG), 8.0 ± 1.5% (30% EMG), and 6.7 ± 1.2% (50% EMG). The ratio at rest was significantly higher than that at all muscle contraction levels (p < 0.05), and no significant differences were observed among the different muscle contraction levels. The increase ratios with ICF were 41.5 ± 9.4% (rest), 12.5 ± 2.8% (10% EMG), 6.1 ± 1.8% (30% EMG), and 4.9 ± 1.2% (50% EMG). The ratio at rest was significantly higher than that at 30% and 50% EMG (p < 0.05), and no significant differences were observed among the different muscle contraction levels.

**Figure 7:**
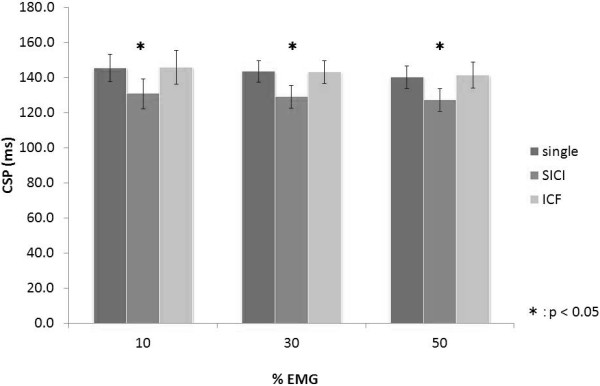
**Histograms of the mean CSP durations with TMS at varying muscle contraction levels.** At all muscle contraction levels, the duration of CSP after SICI was significantly shorter than that induced by single-pulse TMS and after ICF (p < 0.05). No significant difference in CSP duration was observed between single-pulse TMS and ICF.

Results of one-way ANOVA showed that CSP duration changed significantly with paired-pulse TMS (10% EMG, F_(1.476,14.756)_ = 12.734, p < 0.01; 30% EMG, F_(1.988,19.883)_ = 15.789, p < 0.01; 50% EMG, F_(1.801,18.011)_ = 24.969, p < 0.01; Figure 7). CSP duration at 10% EMG was 145.5 ± 7.9 ms (single-pulse TMS), 130.8 ± 8.6 ms (SICI), and 145.9 ± 9.6 ms (ICF). CSP duration at 30% EMG was 143.5 ± 6.1 ms (single-pulse TMS), 129.1 ± 6.5 ms (SICI), and 143.1 ± 6.4 ms (ICF). CSP duration at 50% EMG was 140.1 ± 6.5 ms (single-pulse TMS), 127.3 ± 6.5 ms (SICI), and 141.4 ± 7.4 ms (ICF). At all muscle contraction levels, CSP shortened significantly with SICI compared with single-pulse TMS and ICF (p < 0.05). However, no significant differences were observed in CSP duration between single-pulse TMS and ICF.

**Figure 8:**
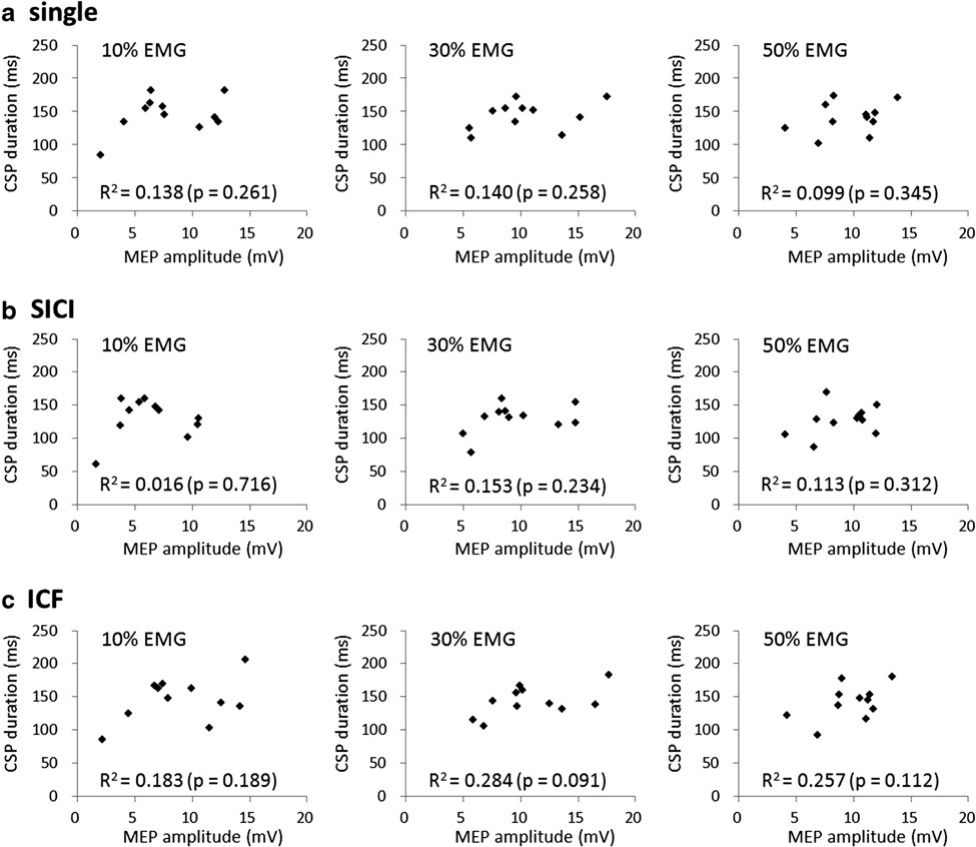
**Relationships between MEP and CSP.** No significant correlations were observed between MEP amplitude and CSP duration under any of the conditions.

No significant correlations were observed between MEP amplitude and the duration of CSP induced by single-pulse TMS and after SICI and ICF at 10%, 30%, and 50% EMG (single-pulse TMS, p = 0.261, 0.140, and 0.345; SICI, p = 0.716, 0.234, and 0.312; ICF, p = 0.189, 0.091, and 0.112, respectively; Figure 8).

## Discussion

We confirmed that CSP duration is affected by TMS intensity but not by the muscle contraction level or MEP amplitude. Our new findings were that CSP is shortened with SICI, but it is not altered by ICF, when the TMS intensity is constant. These results indicate that after SICI, CSP duration is affected by the activity of inhibitory intermediate neurons that are activated by the conditioning SICI stimulus.

### Effects of muscle contraction on MEP amplitude and CSP duration (experiment 1)

The MEP amplitude increased with an increase in muscle contraction intensity from 10% to 50% EMG in the present study. This result is in agreement with the results of previous reports [7,27,28]. However, we did not observe an increase in MEP amplitude above 50% EMG despite the increase in MEP amplitude due to an increase in muscle contraction intensity. The most motor units of the first dorsal interosseous (FDI) muscle are recruited below 50% MVC [29], whereas the maximum recruitment threshold of other large muscles is 95% or 90% MVC [30]. We consider that an increase in MEP amplitude in a muscle contraction state above 50% EMG could not be observed because most of the FDI motor units had already been recruited.

In contrast, CSP duration was always constant and was not influenced by muscle contraction intensity. CSP results from the activity of inhibitory intermediate neurons attached to pyramidal cells of the motor cortex [4,6]. Because the stimulation threshold that induces CSP is of a lower intensity than that which induces MEP [31,32], inhibitory intermediate neurons that generate CSP and pyramidal cells involved in generating MEP through stimulation at 100% AMT can be stimulated [33]. The results of our experiment show that although changes in cortical excitability as a result of muscle contraction have an effect on the excitability of pyramidal cells for MEP generation, they have no effect on the inhibitory intermediate neurons that induce CSP. It has been reported that high MEP amplitude induces a long CSP [6], but the results of experiment 1 support reports that MEP amplitude and CSP fluctuate independently [4,7,10,11,34-36].

### Effects of TMS intensity on MEP amplitude and CSP duration (experiment 2)

We did not observe an increase in MEP above a stimulation intensity of 140% despite the MEP amplitude increasing with an increase in TMS intensity. Because many motor units are already excited at a stimulation intensity above 140% AMT, we may not have observed a further increase. Nonetheless, CSP was prolonged with TMS intensity increase from 100% AMT to 160% AMT. These results support the reports by Orth et al. [33] and Kimiskidis et al. [37] and are considered to show that CSP is not related to muscle contraction intensity or MEP amplitude, but it varies depending on magnetic stimulation intensity.

### Effects of paired-pulse TMS on MEP amplitude and CSP duration (experiment 3)

SICI and ICF were also observed at 10% and 30% EMG, similar to SICI and ICF at rest. In contrast, although SICI was observed at 50% EMG, ICF was not. Ilić TV et al. [19]reported that SICI was observed with paired-pulse stimulation during approximately 5%–10% muscle contractions, whereas ICF was attenuated compared with ICF at rest. In the present experiment, the facilitation effect as a result of ICF was attenuated with increasing muscle contraction intensity from 10% to 50% EMG. ICF disappeared at 50% EMG, but many motor units were already in a mobilized state at 50% EMG, similar to the results in experiment 1, which could explain why no ICF occurred.

In contrast, SICI was observed even at an intensity of 50% EMG, despite the inhibitory effect resulting from SICI attenuation with an increase in muscle contraction intensity. Ortu et al. [25] measured MEP resulting from paired-pulse stimulation at 10%, 25%, and 50% MVC and reported observing SICI at 10% MVC but not at 25% or 50%. That study was conducted at an intensity of test stimuli that induced a 1 mV MEP amplitude at each muscle contraction intensity. The present study may have produced different results because the magnetic stimulation intensity that induced 1 mV at rest was used at all muscle contraction levels.

CSP was significantly shortened with SICI compared with single-pulse TMS and ICF at all muscle contraction intensities, whereas no significant differences in CSP duration were observed between ICF and single-pulse TMS. A few studies have reported the modulation of CSP by paired-pulse TMS [22-24], but the results have not been consistent. In previous studies, GABAA receptors were reported to be involved in the SICI mechanism [14,17,19,20]. CSP is reported to occur when pyramidal cells in the cortex that sustain muscle contractions are suppressed [25]. However, GABAA-mediated inhibitory interneurons were excited because of the conditioning pulse of SICI and because the activity of inhibitory intermediate neurons that cause CSP and the proliferation of pyramidal cells involved in MEP were suppressed, CSP was shortened. There are many uncertainties about the action mechanism of ICF. Di Lazzaro et al. [21] reported that ICF is a change caused by the excitability of the spinal cord, whereas Nakamura et al. [15] suggested the possibility of facilitation within the cortex. Our present experiment did not clarify whether the influences of the intracortical or subcortical network cause ICF. However, under conditions in which ISI of paired-pulse stimulation was 10 ms, it was clear that this did not change CSP, although MEP was increased.

## Conclusions

This study investigated the effects of muscle contractions and stimulus intensities on MEP and CSP induced by single-pulse TMS and the effects of SICI and ICF on CSP duration. Our major results were that CSP is affected by TMS intensity but not by the muscle contraction level or MEP amplitude. In addition, although TMS intensities were constant, CSP was significantly shortened with SICI compared with single-pulse TMS and ICF. These results indicated that after SICI, CSP is affected by the activity of inhibitory intermediate neurons that were activated by the conditioning SICI stimulus.

## Methods

### Subjects

Fifteen healthy, right-handed, male volunteers (age, 21–43 years; mean ± SD, 25.1 ± 5.1 years) participated in this study. All subjects gave their written informed consent. This study was approved by the ethics committee at the Niigata University of Health and Welfare, Niigata, Japan (17279–111012).

### EMG recording

The subjects were seated comfortably in a chair. EMGs were recorded from the right FDI muscle using a silver/silver-chloride electrode in a belly tendon montage. EMG signals were amplified (×1000) by an amplifier (A-DL-720•140; 4 Assist, Tokyo, Japan) and digitized at 2 kHz using a A/D converter (PowerLab 8/30; AD Instruments, CO, USA). The subjects performed right index finger abduction, and MVC and maximum EMG values of FDI were measured (100% EMG). The EMG signals were rectified and smoothed with a 501-point smoothing. The muscle contraction level was controlled by displaying a constantly updated bar graph showing the smoothed EMG amplitude (expressed as a percentage of maximal contraction). These monitors also allowed us to confirm the state of muscle contraction. The subjects were instructed to contract and sustain the targeted levels at 0 (rest), 10, 30, 50, and 70% EMG.

### TMS

Monophasic pulse TMS was delivered with a figure-of-eight-shaped coil (diameter, 95 mm) connected to Magstim 200 (Magstim, Dyfed, UK). The coil was held with the handle pointing backward and laterally at approximately 45° to the sagittal plane. The optimal spot to elicit MEP in the right FDI was carefully determined in each subject, and the optimal coil position to evoke a stable MEP was marked on the cap worn by the subject. AMT was obtained during a slight isometric contraction (approximately 5% MVC) and was defined as the lowest stimulus intensity able to induce MEP with greater than 100 μV peak-to-peak amplitude in FDI in at least 5 of 10 consecutive trials [14]. The 1 mV TMS intensity was defined as the lowest stimulus intensity able to induce MEP with greater than 1 mV peak-to-peak amplitude in the relaxed FDI in at least 5 of 10 consecutive trials [25].

### Experiment 1 (effects of muscle contraction on MEP amplitude and CSP duration)

**Figure 9:**
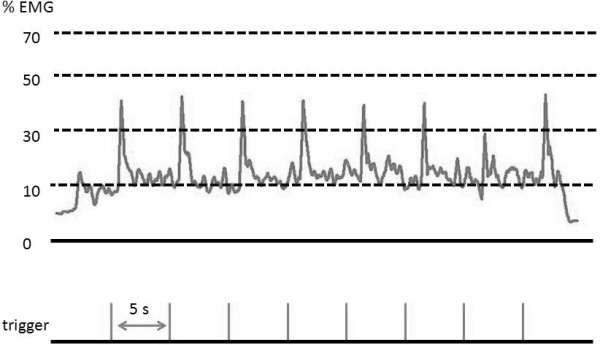
**TMS trigger and rectified and smoothed EMG during sustained 10% EMG in experiment 1.** ISI was set at 5 s in all experiments. In experiment 1, stimulus intensity was set at 100% AMT, and the subjects maintained the target EMG in isolation at 10%, 30%, 50%, and 70% EMG. In experiment 2, the subjects were stimulated at intensities of 100%, 120%, 130%, 140%, 150%, and 160% AMT during sustained 10% EMG. In experiment 3, single- and paired-pulse TMS with ISIs of 3 and 10 ms were delivered at 0, 10, 30, and 50% EMG.

The effect of muscle contraction strength on MEP and CSP induced by single-pulse TMS was investigated in 11 subjects (age, 23.3 ± 2.2 years). The subjects sustained the target EMG in isolation at 10%, 30%, 50%, and 70% EMG for 40 s. TMS intensity was 100% AMT, and 8 stimuli were applied at 0.2 Hz. A representative TMS trigger and rectified and smoothed EMG during sustained 10% EMG in experiment 1 are shown in Figure 9.

### Experiment 2 (effects of TMS intensity on MEP amplitude and CSP duration)

We investigated the effect of stimulus intensity on MEP and CSP in the same 11 subjects as in experiment 1. Right index finger abduction was performed in the same manner as in experiment 1. The muscle contraction level was 10% EMG, and TMS intensity was set at 100%, 110%, 120%, 130%, 140%, 150%, and 160% AMT with 0.2 Hz frequency. Muscle contraction was sustained for 40 s, and eight stimuli were applied at each TMS intensity level.

### Experiment 3 (effects of paired-pulse TMS on MEP amplitude and CSP duration)

The effects of SICI and ICF induced by paired-pulse TMS on MEP amplitude and CSP duration were investigated in 11 subjects (age, 27.1 ± 8.2 years, including seven of the same subjects as in experiment 1). The stimulus intensities used for paired-pulse TMS were 80% AMT as a conditioning pulse and 1 mV TMS intensity as the test pulse. These conditioning and test intensities were the same for all muscle contraction levels, because CSP duration might be influenced by TMS intensity [33]. ISI of the paired-pulse TMS was 3 ms (SICI) and 10 ms (ICF). SICI and ICF were recorded using three stimulations, including single-pulse TMS, at rest, and three contractions (10%, 30%, and 50% EMG). Paired-pulse TMS of each ISI and single-pulse TMS were randomly presented among a total of 24 stimuli and were applied at 0.2 Hz.

### Data analysis

MEP amplitudes were calculated from peak-to-peak amplitudes, except for the amplitude of the maximum and minimum MEP amplitude of 8 waves. CSP duration was defined as the time from TMS onset to the time of reappearance of EMG amplitude that was more than 3-fold the SD of the background EMG noise at rest, and the average values were calculated for all CSP durations. The decrease and increase ratios with SICI and ICF, respectively, [absolute (conditioned MEP − unconditioned MEP)/unconditioned MEP × 100) were calculated at each muscle contraction level.

Statistical analysis was performed using PASW statistics 18 software (IBM SPSS, Armonk, NY, USA). All data, including MEP amplitudes and CSP durations, were statistically analyzed by one-way repeated measures ANOVA with muscle contractions, stimulus intensities, and ISI as the within-subject factors. The sphericity of data was analyzed by Mauchly’s test, and Greenhouse–Geisser-corrected significance values were used when sphericity was lacking. Post hoc analysis was performed with Bonferroni’s methods for multiple comparisons to avoid type I errors. The correlations between MEP amplitude and the duration of CSP induced by single-pulse TMS and after SICI and ICF at each muscle contraction level were assessed by Pearson’s correlation analysis. Differences were considered significant at p < 0.05 for all analyses.

## Abbreviations

CSP: Cortical silent period; TMS: Transcranial magnetic stimulation; MEP: Motor evoked potential; SICI: Short-interval intracortical inhibition; ICF: Intracortical facilitation; AMT: Active motor threshold; EMG: Electromyogram; SD: Standard deviation; SEM: Standard error of the mean; ISI: Interstimulus intervals; MVC: Maximum voluntary contraction; ANOVA: Analysis of variance; FDI: First dorsal interosseous.

## Competing interests

The authors declare no competing interests.

## Authors’ contributions

SK conceived of the study, designed the experimental paradigm, performed the statistical analysis, and contributed to the discussion and preparation of the manuscript. HO conceived of the study, designed the experimental paradigm, performed the statistical analysis, and contributed to the discussion and preparation of the manuscript. KS conceived of the study, designed the experimental paradigm, performed the statistical analysis, and contributed to the discussion and preparation of the manuscript. HK conceived of the study, designed the experimental paradigm, and contributed to the discussion of the manuscript. MS performed the statistical analysis and contributed to the discussion and preparation of the manuscript. HT conceived of the study, designed the experimental paradigm, and contributed to the discussion of the manuscript. All authors read and approved the final manuscript.
